# Combining Digital Watermarking and Fingerprinting Techniques to Identify Copyrights for Color Images

**DOI:** 10.1155/2014/454867

**Published:** 2014-07-08

**Authors:** Shang-Lin Hsieh, Chun-Che Chen, Wen-Shan Shen

**Affiliations:** ^1^Department of Computer Science and Engineering, Tatung University, Taipei 10452, Taiwan; ^2^Taipei College of Maritime Technology, New Taipei 25172, Taiwan

## Abstract

This paper presents a copyright identification scheme for color images that takes advantage of the complementary nature of watermarking and fingerprinting. It utilizes an authentication logo and the extracted features of the host image to generate a fingerprint, which is then stored in a database and also embedded in the host image to produce a watermarked image. When a dispute over the copyright of a suspect image occurs, the image is first processed by watermarking. If the watermark can be retrieved from the suspect image, the copyright can then be confirmed; otherwise, the watermark then serves as the fingerprint and is processed by fingerprinting. If a match in the fingerprint database is found, then the suspect image will be considered a duplicated one. Because the proposed scheme utilizes both watermarking and fingerprinting, it is more robust than those that only adopt watermarking, and it can also obtain the preliminary result more quickly than those that only utilize fingerprinting. The experimental results show that when the watermarked image suffers slight attacks, watermarking alone is enough to identify the copyright. The results also show that when the watermarked image suffers heavy attacks that render watermarking incompetent, fingerprinting can successfully identify the copyright, hence demonstrating the effectiveness of the proposed scheme.

## 1. Introduction

Many researchers [1–17] have been engaged in finding the solution to protecting copyrights of digital images, which may be duplicated and distributed over the Internet without the authors' permission. Generally speaking, there are two approaches to discovering image copyright infringement. One is watermarking [1–11] and the other is fingerprinting [12–17]. The main idea of watermarking is to embed a piece of information (i.e., watermark) in the host image. If a similar watermark can be retrieved from a suspect image, it is then considered a duplicated one. On the other hand, the principle of fingerprinting is to extract unique features (i.e., fingerprints) from both the host image and the suspect one for comparison. If their fingerprints are similar, the ownership of the image can then be confirmed.

There are some general considerations on the two techniques, including the processing time and robustness. In terms of processing time, watermarking is more efficient because fingerprinting needs extra time to compare the image's fingerprint with those stored in the database. If the database is large, it will be very time consuming. On the other hand, fingerprinting is generally more robust [18, 19] because when a watermarked image suffers some image processing operations that modify the content of the image, the embedded watermark will usually be damaged or even destroyed. On the contrary, since normal image processing does not destroy the features of an image, the fingerprint of the image can therefore be preserved. In summary, fingerprinting is more robust whereas watermarking is more efficient. If the complementary natures of two approaches can be utilized properly, a robust and efficient scheme can then be developed to identify copyrights.

This paper proposes a novel scheme that combines the two techniques to identify copyrights for color images. The proposed scheme generates from the image a fingerprint, which also serves as the watermark. The watermark is then embedded in the host image to produce a watermarked image. When there is a dispute over the copyright of a suspect image, the suspect image will first be processed by watermarking, which tries to retrieve the watermark from the suspect image. If the watermark is identified, the copyright is confirmed at this stage; otherwise, the image will then be processed by fingerprinting, which utilizes the retrieved watermark as the fingerprint and compares it with those stored in the database. If a match is found, then the suspect image will be considered a duplicated one.

## 2. Related Background

The proposed scheme utilizes a special technique called image secret sharing (ISS), whose details can be found in the paper [20] we published in 2008. The following briefly describes the main idea of the technique utilized by the proposed scheme.

**Figure 1:**
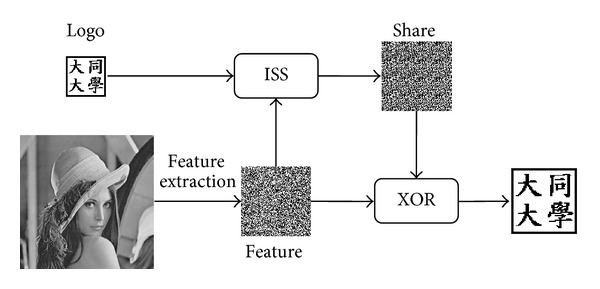
The process of the ISS.

**Figure 2:**
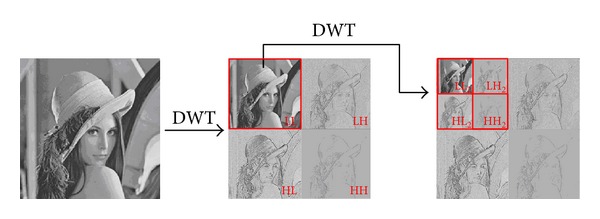
2D DWT.

The ISS generates a share image from two images. In the proposed scheme, the two images are the logo image and the feature image (as depicted in Figure 1). The logo image can be any identifiable image. The feature image is generated from the input image as follows. First, the input image is split into nonoverlapping 8 × 8 blocks. Then, the 2D DWT is applied to each block to generate four subbands, LL_2_, LH_2_, HL_2_, and HH_2_. An example of 2D DWT is shown in Figure 2. Finally, the coefficients in the LL_2_ subband of each DWT block are used to generate the feature image. The ISS then generates a share image from the logo image and feature image. The share image will be used as the fingerprint of the input image by the scheme. The share image also serves as the watermark to be embedded in the host image. The benefit of the ISS scheme is that performing the XOR operation on the feature image and the share image will restore the logo image, which can then be used to identify the copyright.

## 3. The Proposed Copyright Identification Scheme

The proposed scheme contains two phases: the* fingerprint and watermarked image generation* phase and the* authentication logo detection* phase. The former phase extracts features from the host image, which, along with a logo image, is used to generate the fingerprint. The fingerprint also serves as the watermark, and the phase embeds it in the host image to produce a watermarked image. On the other hand, the latter phase extracts features and retrieves the watermark from the suspect image. The extracted features and the retrieved watermark are utilized to restore the logo image, which is used to identify the copyright. If it fails, the retrieved watermark then serves as the fingerprint and is compared with those in the database to determine if the suspect image is a duplicated one.

**Figure 3:**
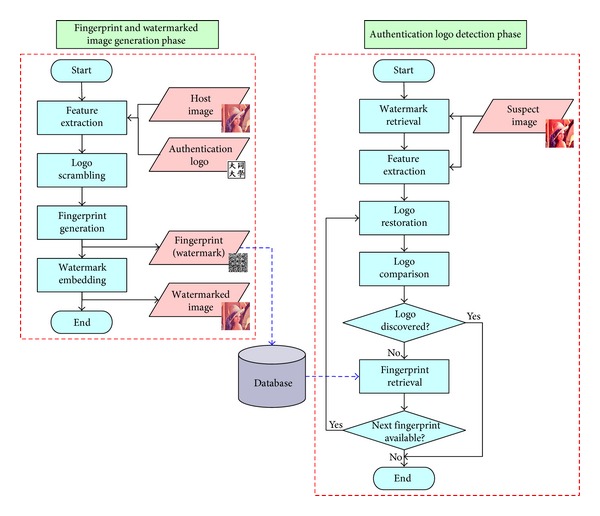
The phases of the proposed scheme.

The* fingerprint and watermarked image generation* phase (shown in Figure 3) works as follows. In the beginning,* feature extraction* extracts the features of the host image and then* logo scrambling* disarranges the authentication logo to a scrambled logo image. After that,* fingerprint generation* takes as input the extracted features and the scrambled logo to generate the fingerprint. Finally, the fingerprint serves as a watermark and is embedded in the host image, which becomes a watermarked image. The fingerprint is also stored in a database for later use in the next phase.

The* authentication logo detection* phase (shown in Figure 3) checks the watermark first and, if necessary, the fingerprint next. In the beginning,* watermark retrieval* regains the watermark from the suspect image. Next, the features of the suspect image are extracted by* feature extraction*. After that,* logo restoration* takes as input the retrieved watermark (the expected fingerprint of the suspect image) and the extracted features to recover and rearrange the scrambled logo to restore the authentication logo. The phase ends if the accuracy rate of the restored logo determined by* logo comparison* is high enough; otherwise, the process proceeds to retrieve the next available fingerprint from the database and then returns to* logo restoration*, which takes as input the retrieved fingerprint instead of the extracted watermark. The phase restores the logo from the retrieved fingerprint as well as the extracted features and proceeds to* logo comparison*. The looping process continues until the authentication logo is discovered or no fingerprint is available.

### 3.1. Fingerprint and Watermarked Image Generation Phase

The following paragraphs detail the stages in the* fingerprint* and* watermarked image generation* phase, including* feature extraction, logo scrambling, fingerprint generation,* and* watermark embedding.*

#### 3.1.1. Feature Extraction

**Figure 4:**
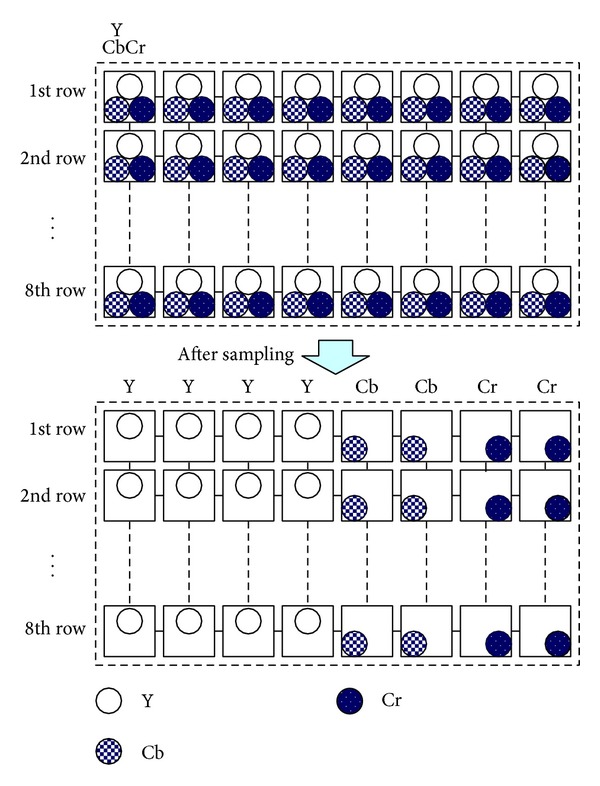
Illustration of* sampling*.

The* feature extraction* stage takes a color image as input and then extracts its features. The stage has two substages,* sampling* and* feature generation*. During* sampling*, the stage first transforms the RGB image to the YCbCr color space [21, 22]. Then, it partitions each of the three channels into several nonoverlapping blocks of size 8 × 8 (hence, a color image of size *N* × *N* will result in 3 × *N*/8 × *N*/8 nonoverlapping blocks). After partition, for each row of the corresponding blocks in the three channels, the stage takes the first four samples from the Y channel, the next two from the Cb, and the last two from the Cr (as depicted in Figure 4) to generate new packed blocks, each of size 8 × 8.

After* sampling*, the stage enters its second substage,* feature generation*. For each of the packed blocks, the stage applies 2D DWT to the block, resulting in four coefficients in the LL_2_ subband. Next, the stage computes the average (denoted by *A*) of the four coefficients and then obtains a feature type *T* according to the relationship of the four coefficients and the average *A* as expressed in (1). Consider

(1) $$\begin{array}{c} T=\{\begin{array}{cc} 1, & \text{if}\;\;\text{only}\;\;\text{one}\;\;\text{coefficient}\;\;\text{is}\;\;\text{smaller}\;\;\text{than}\;\;A.\\ 2, & \text{if}\;\;\text{only}\;\;\text{two}\;\;\text{coefficients}\;\;\text{are}\;\;\text{smaller}\;\;\text{than}\;\;A.\\ 3, & \text{if}\;\;\text{only}\;\;\text{one}\;\;\text{coefficient}\;\;\text{is}\;\;\text{greater}\;\;\text{than}\;\;A.\\ 4, & \begin{array}{c} \text{if}\;\;\text{all}\;\;\text{of}\;\;\text{the}\;\;\text{four}\;\;\text{coefficients}\;\;\text{are}\;\;\text{the}\\ \text{same}\;\;\text{and}\;\;\text{hence}\;\;\text{all}\;\;\text{equal}\;\;A. \end{array} \end{array} \end{array}$$

According to *T*, *A*, and the mapping table shown in Table 1, a feature share (called FT-share) of size 2 × 2 is determined for each block. The FT-shares represent the features of the input color image. They are assembled to form the feature image.

The steps of the* feature extraction* stage are listed in Algorithm 1.

#### 3.1.2. Logo Scrambling

**Figure 5:**
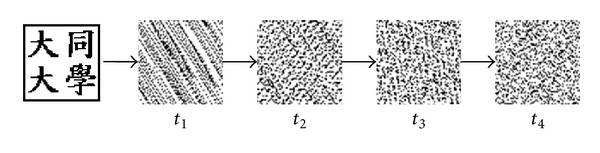
Example of scrambling.

In order to disperse the intensity of attacks, the proposed scheme adopts Torus automorphism [23] to scramble the authentication logo. The stage uses a predetermined key, *k*, and the following equation to scramble the logo. Consider

(2) $$\begin{array}{c} \left(\begin{array}{c} x_{t}\\ y_{t} \end{array}\right)=\left(\begin{array}{cc} 1 & 1\\ k & k+1 \end{array}\right)\left(\begin{array}{c} x_{t-1}\\ y_{t-1} \end{array}\right)\mathrm{mod}T, \end{array}$$

where (*x*_*t*_, *y*_*t*_) are the coordinates in state* t* and *T* is the coordinate size of the given image.  Figure 5 shows an example of the authentication logo scrambled four times with *k* = 1, *T* = 128.

#### 3.1.3. Fingerprint Generation

The proposed scheme uses ISS (mentioned in Section 2) to generate the fingerprint. It determines a FP-share for each FT-share (generated in* feature extraction*) according to the color of the corresponding pixel in the scrambled logo by looking Table 1 up. For example, if the feature type is 2 and the FT-share is the same as the one in Table 2, then the FP-share will be either one of the two FP-shares in Table 2 according to the color of the corresponding pixel in the scrambled logo. After every FP-share is determined, the stage gathers all the FP-shares to form the fingerprint image. The fingerprint also serves as the watermark in the next stage.

#### 3.1.4. Watermark Embedding

**Figure 6:**
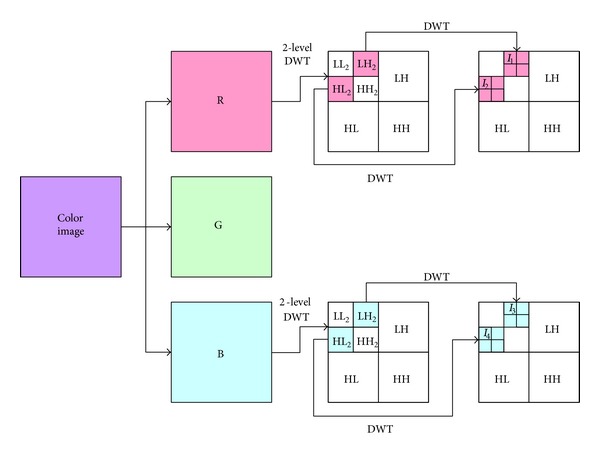
The *I*_1_, *I*_2_, *I*_3_, and *I*_4_ blocks used for* watermark embedding*.

The* watermark embedding* stage uses the resulting fingerprint image as a watermark and then embeds it in the host image. Generally speaking, for the RGB color space, the human visual system is more sensitive to the G channel than to the other two [22–28]. Therefore, the proposed scheme embeds the watermark into the less sensitive R and B channels of the host image. To be more specific, there are four areas: two in the R channel (*I*_1_ and *I*_2_) and two in the B channel (*I*_3_ and *I*_4_), used for* watermark embedding* (see Figure 6). The stage applies the 2D DWT to the R and B channels of the host image and next applies the 1D DWT to the resulting HL_2_ and LH_2_ to obtain the four blocks, *I*_1_, *I*_2_, *I*_3_, and *I*_4_.

**Figure 7:**
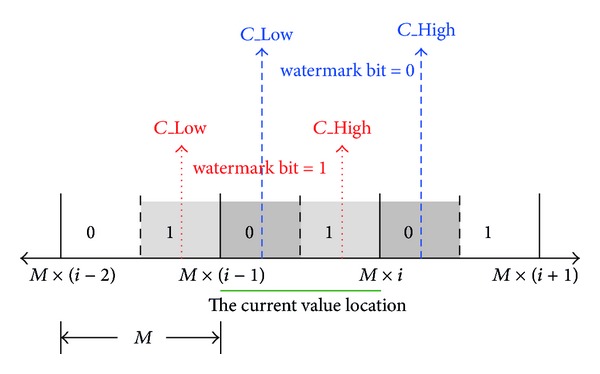
Coefficient adjustment.

The proposed scheme embeds the watermark into the image by adjusting the coefficients in the *I*_1_ to *I*_4_ blocks according to a predefined *M*. The value of *M* affects the robustness and the quality. The larger the *M* is, the more robust the embedded watermark is, but the worse the quality of the watermarked image is.  Figure 7 illustrates the adjustment of the coefficients. If the watermark bit is 1 and the current coefficient is between *M* × (*i* − 1) and *M* × *i*, then the coefficient will be adjusted to be *M* × (*i* − 1) − *M*/4 or *M* × *i* − *M*/4, whichever is closer to the current coefficient. On the other hand, the adjusted coefficient will be *M* × (*i* − 1) + *M*/4 or *M* × *i* + *M*/4 if the watermark bit is 0.

The embedding process is described as follows. First, the stage uses the current coefficient *c* and the predefined *M* to calculate* S*, sign, and (*r*_0_, *r*_1_) according to (3), (4), and (5), respectively. In the equations, sign indicates that the *c* is positive or negative; *r*_0_ is the remainder for watermark bit value 0 and, similarly, *r*_1_ for watermark bit value 1. Then, the stage determines the* C_*Low and* C_*High according to the value of the watermark bit. If the value is 0, (6) will be used, otherwise, (7). Finally, the stage adjusts the coefficient *c* to *c*′ according to (8). Consider

(3) $$\begin{array}{c} S=\frac{\left|c\right|}{M}, \end{array}$$

(4) $$\begin{array}{c} \mathrm{sign}=\{\begin{array}{cc} 1, & \text{if}\;\;c\geq 0,\\ -1, & \text{otherwise}, \end{array} \end{array}$$

(5) $$\begin{array}{c} \left(r_{0},r_{1}\right)=\{\begin{array}{ccc} \left(\frac{M}{4},\frac{3M}{4}\right), & \text{if}\;\;c\geq 0, & \\ \left(\frac{3M}{4},\frac{M}{4}\right), & \text{otherwise}, & \end{array} \end{array}$$

(6) C_Low=sign⁡×(S×M+r0),C_High=sign⁡×((S+sign⁡)×M+r0),

(7) C_Low=sign⁡×((S−sign⁡)×M+r1),C_High=sign⁡×(S×M+r1),

(8) $$\begin{array}{c} c^{\prime }=\{\begin{array}{cc} C\_\text{Low}, & \text{if}\;\;\left|C\_\text{Low}-c\right|\leq \left|C\_\text{High}-c\right|\\ C\_\text{High}, & \text{otherwise}. \end{array} \end{array}$$

The steps of the* watermark embedding stage* are listed in Algorithm 2.

### 3.2. Authentication Logo Detection Phase

The phase is activated when a dispute over the copyright of a suspect image occurs. The following details the stages in the phase, including* watermark retrieval* and* logo restoration.*

#### 3.2.1. Watermark Retrieval

The stage regains the watermark from the suspect image. First, the stage applies the DWT to the R and B planes of the suspect image in the same way as that in* watermark embedding* to obtain the embedding blocks, *I*_1_ to *I*_4_ (refer to Figure 6). Then, for each coefficient *c* of the *I*_1_ *–I*_4_blocks, it obtains the watermark bit *w* according to

(9) $$\begin{array}{c} w=\{\begin{array}{cc} 1, & \begin{array}{cc} \text{if}\;\;\left(c\geq 0,\;\;c\mathrm{mod}M\geq \frac{M}{2}\right), & \\ \text{or}\;\;\left(c<0,\;\;\left|c\right|\mathrm{mod}M<\frac{M}{2}\right), & \end{array}\\ 0, & \text{otherwise}, \end{array} \end{array}$$

where *M* is the same as that in (3).

Finally, the watermark (which is supposed to be the fingerprint of the image) is restored by assembling every *w* for each coefficient *c* in the *I*_1_–*I*_4_ blocks.

Algorithm 3 lists the steps of the* watermark retrieval* stage.

#### 3.2.2. Logo Restoration

**Figure 8:**
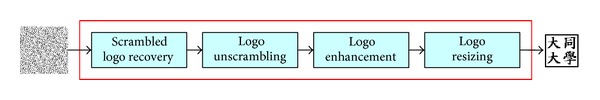
The four substages of* logo restoration*.

The stage restores the authentication logo. As shown in Figure 8, it has four substages:* scrambled logo recovery*,* logo unscrambling*,* logo enhancement*, and* logo resizing*.

*Scrambled Logo Recovery.* After retrieving the fingerprint image (i.e., watermark) by* watermark retrieval* and extracting the feature image by* feature extraction*, the substage performs XOR operation on each pixel of the fingerprint image and the corresponding pixel of the feature image to retrieve the scrambled logo. Because both of the two images are black-and-white, each pixel is either 0 (black) or 1 (white). Therefore, the substage simply performs bitwise XOR operation on the two images and obtains a scrambled logo.

*Logo Unscrambling*. As mentioned in Section 3.1.2, the logo was scrambled before it is used to generate fingerprint in the* fingerprint and watermarked image generation* phase. The substage adopts (10), which is the inverse equation of (2), to rearrange the scrambled logo and restore the logo. Consider

(10) $$\begin{array}{c} \left(\begin{array}{c} x_{t}\\ y_{t} \end{array}\right)={\left(\begin{array}{cc} 1 & 1\\ k & k+1 \end{array}\right)}^{-1}\left(\begin{array}{c} x_{t-1}\\ y_{t-1} \end{array}\right)\mathrm{mod}T. \end{array}$$

**Figure 9:**
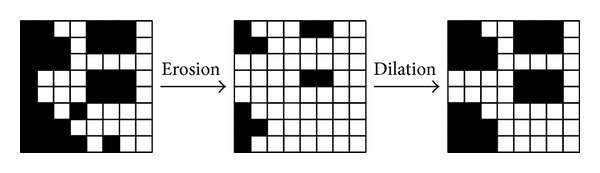
An example of erosion and dilation.

*Logo Enhancement.* The substage enhances the restored logo by erosion and dilation. Erosion removes pixels on object boundaries in an image and therefore can be used to remove smaller islands in the image; dilation, on the other hand, adds pixels to the boundaries of objects in an image and hence can be used to remove bright areas from the image. The substage performs erosion followed by dilation once, which is illustrated in Figure 9.

Erosion is performed by resetting each of the pixels according to (11). That is, if one of the neighbors is black, then the current pixel will be set to black; otherwise, it will be set to white. Let* L*(*x*,* y*) be the current pixel value to be determined and *L*(*x* + 1, *y*), *L*(*x* + 1, *y* + 1), and *L*(*x*, *y* + 1) its neighbors; then

(11) $$\begin{array}{c} L\left(x,y\right)=\{\begin{array}{cc} 0\left(\text{black}\right), & \begin{array}{c} \text{if}\;\;L\left(x+1,y\right)\;\;\text{or}\;\;L\left(x+1,y+1\right)\\ \text{or}\;\;L\left(x,y+1\right)\;\;\text{is}\;\;\text{black} \end{array}\\ 1\left(\text{white}\right), & \text{otherwise}. \end{array} \end{array}$$

Dilation, on the contrary, resets the neighbors of each pixel rather than the pixel itself. If* L*(*x*,* y*) is black; the substage sets all of its neighbors, *L*(*x* + 1, *y*), *L*(*x* + 1, *y* + 1), and *L*(*x*, *y* + 1), to black; otherwise, the neighbors remain unchanged.

*Logo Resizing.* The proposed scheme adopts ISS to retrieve the authentication logo, which causes* pixel expansion* because one logo pixel is mapped to a share of four pixels (mentioned in Section 3.1.3). As a result, the retrieved logo from the previous stage will be larger than the original one. To resize the logo to its original size, the substage partitions the enhanced logo into several blocks of size 2 × 2, each of which is then reduced into one pixel with value *L*(*x*, *y*) according to the following rule:

(12) $$\begin{array}{c} L\left(x,y\right)=\{\begin{array}{cc} 0\left(\text{black}\right), & \begin{array}{c} \text{the}\;\;\text{number}\;\;\text{of}\;\;\text{the}\;\;\text{black}\;\;\text{pixels}\\ \text{in}\;\;\text{each}\;\;\text{block}\geq 3, \end{array}\\ 1\left(\text{white}\right), & \text{otherwise}. \end{array} \end{array}$$

## 4. Experimental Results

**Figure 10:**
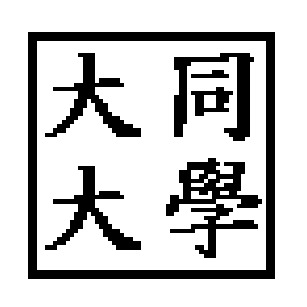
The authentication logo (64 × 64).

Two kinds of experiments were conducted to prove the effectiveness of the proposed scheme. The first experiment shows the robustness of our scheme and the other demonstrates the capability of unique identification. In the experiments, the authentication logo used to generate the watermark (fingerprint) is shown in Figure 10.

Two common measurements used to estimate the robustness of our scheme are described as below.

(1)* Peak Signal to Noise Ratio (PSNR).* The measurement to estimate the color image quality after image processing is a variant version of normal PSNR [29]. The variant PSNR listed below does not consider the influence of the green channel because the channel is not modified by our scheme. Consider

(13) $$\begin{array}{c} \text{PSNR}=10\;\text{lo}{\text{g}}_{10}\frac{255^{2}}{\left(\text{MSE}\left(R\right)+\text{MSE}\left(B\right)\right)/2}\text{dB}, \end{array}$$

where MSE is the* mean square error* between the original image and the modified image, which is defined as follows:

(14) $$\begin{array}{c} \text{MSE}=\frac{\sum_{i=1}^{N}\sum_{j=1}^{N}{\left(x_{ij}-x_{ij}^{\prime }\right)}^{2}}{N^{2}}, \end{array}$$

where *x*_*ij*_ represents the original pixel value and *x*_*ij*_′ denotes the modified pixel value.

**Figure 11:**
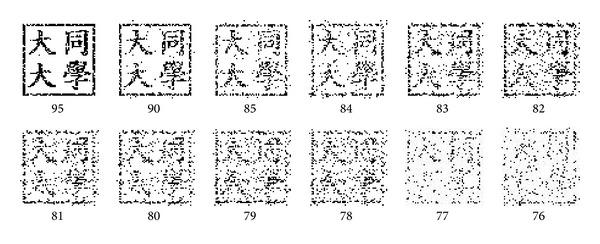
The restored logo with their* AR* values (%).

According to the definition of PSNR, the higher the value is, the better the quality of the modified image is. Generally, if the PSNR is greater than 30 dB, the quality of the modified image is acceptable.

(2)* Accuracy Rate (AR).* The measurement shown below is used to evaluate the correctness of the logo after it has been restored. Consider

(15) $$\begin{array}{c} \text{AR}\left(\%\right)=\frac{\text{CP}}{\text{NP}}\times 100, \end{array}$$

where NP is the number of pixels in the original logo and CP is the number of correct pixels obtained by comparing the pixels of the original logo with the corresponding ones of the restored logo.  Figure 11 shows the restored logos with different AR values. As can been seen, restored logos with AR higher than 81% (the ones in the upper row) still can be visually recognized whereas the restored logo with AR equal to 76% is hard to identify. However, according to the description in Section 4.2, if AR is higher than 75%, the scheme still can identify the copyrights of the image.

### 4.1. Robustness Experiments

**Figure 12:**
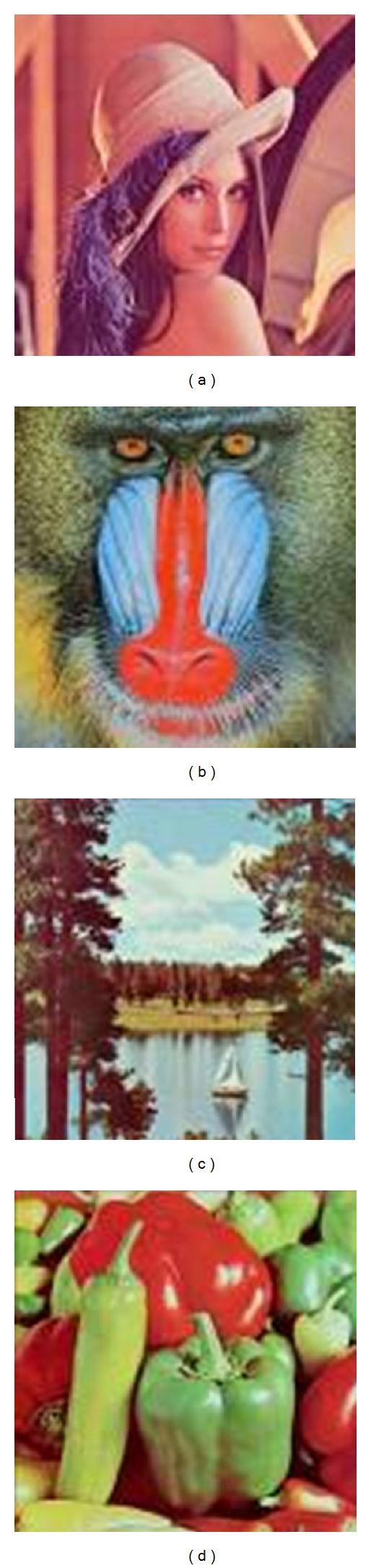
The test images used in the experiments: (a) Lena, (b) Mandrill, (c) Sailboat, and (d) Peppers.

**Figure 13:**
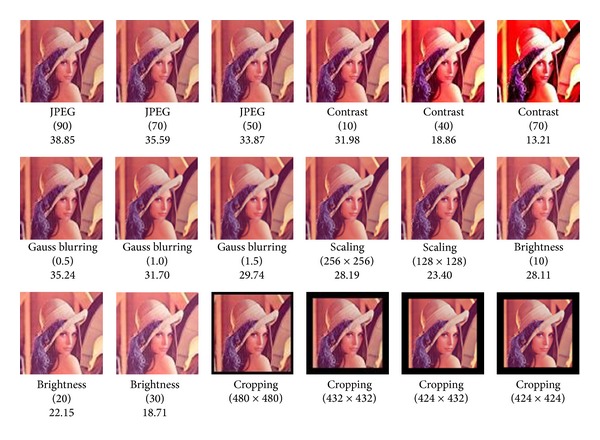
Some examples of "Lena" after different image attacks (PSNRs are listed in the last rows).

The experiments proved our scheme is robust to different kinds of attacks. The test images used in the experiment, including “Lena,” “Mandrill,” “Sailboat,” and “Peppers,” are shown in Figure 12. The commercial image processing software “Adobe Image Photoshop CS” was used to simulate several kinds of image attacks, some of which for “Lena” are shown in Figure 13.

The experimental results of the test images are shown in Table 3. As mentioned above, if AR is less than 75%, the scheme cannot identify the copyrights of the image. Table 3 shows that our scheme failed to verify the copyrights for the images suffering the heavier attacks of JPEG, contrast, Gauss blurring, and scaling in* watermark verification*. Nevertheless, the duplications of those images can all be determined in* fingerprint verification*. In summary, our scheme can identify the copyrights of the suspect images under moderate attacks in* watermark verification* and determine the duplications of those suffering the heavy attacks in* fingerprint verification*.

Moreover, there were 100 attacks in total and 53 of them resulted in an AR value higher than 75% in* watermarking verification*. That is to say, the copyrights of the 53% of the attacked images can be successfully identified in* watermark verification*, and hence only 47% of them need* fingerprint verification*.

### 4.2. Uniqueness Experiments

**Figure 14:**
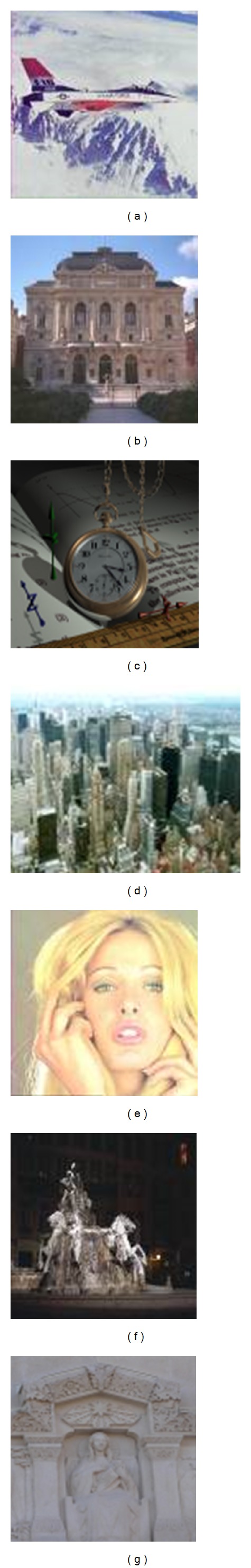
The other seven color images used for testing uniqueness.

The experiment showed that our scheme has the capability of unique identification and is able to distinguish a copyrighted image from different ones. The four copyrighted images (with embedded watermarks) in Figure 12 along with seven unwatermarked images (Figures 14(a)–14(g)) were processed by our scheme to identify the copyright. The stored fingerprints of the watermarked images were used in* fingerprint verification* to restore the logos for all of the images.

The results shown in Table 4 demonstrated the extraordinary unique identification capability of our scheme. It can be clearly seen that all the restored logos except the ones of the copyrighted images (those on the rightmost side) are unrecognizable, which proves that our scheme is actually able to distinguish a copyrighted image from different ones. It will not mistakenly identify the copyrights of an unwatermarked image.

Moreover, the resulting AR values of all the unwatermarked images are all lower than 75%. Hence, it is reasonable for our scheme to confirm the copyright when AR is higher than 75%.

### 4.3. Discussion and Comparison

**Figure 15:**
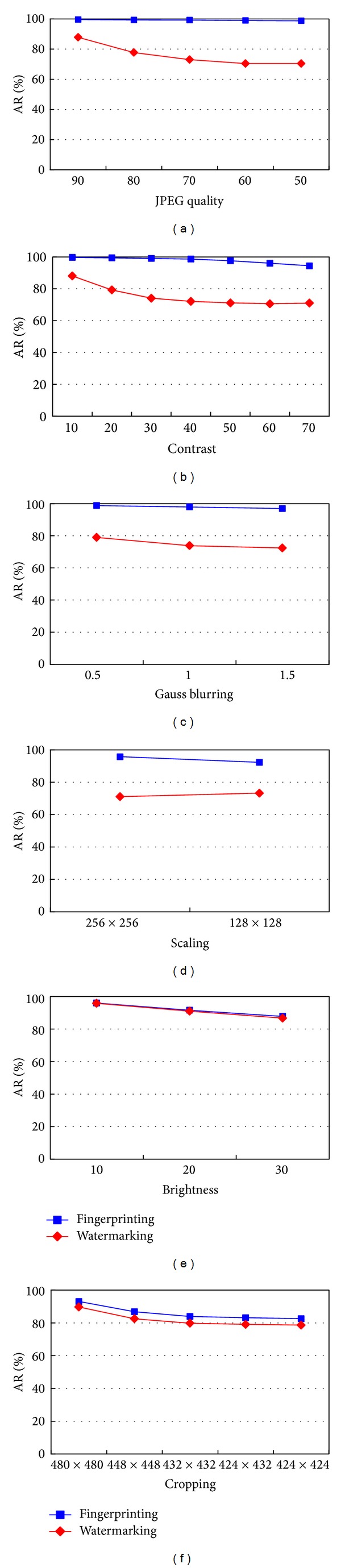
The average* AR* values of test images: (a) JPEG quality, (b) contrast, (c) Gauss blurring, (d) scaling, (e) brightness, and (f) cropping.

The fingerprint extracted from an image is more robust than the watermark embedded in the image. This can be seen from Figure 15, which shows the average AR values of the images suffering different attacks for watermarking and fingerprinting. The AR values for fingerprinting are all higher than those for watermarking. When the image undergoes image processing operations that heavily damage the embedded watermark, the extracted fingerprint still can survive the attacks. However, because linear comparison is a computationally intensive process, fingerprinting is costly in time if there are many fingerprints in the database.

Table 5 shows the processing time for* watermark extraction* and* logo restoration and comparison* in our scheme. The experiments were carried out on a computer equipped with the following hardware and software:

 CPU: 3.16 GHz Intel(R) Xeon(R) CPU E3120,
 RAM: 4 GB,
 OS: Windows 7,
 Computing language and environment: MATLAB.

As Table 5 shows, it takes about 0.21 seconds to extract the watermark from the input image and 0.029 seconds to restore and compare the logo. Therefore, the total processing time *T*_*p*_ (in seconds) of our scheme can be expressed by

(16) $$\begin{array}{c} T_{p}=0.24+0.029\times n, \end{array}$$

where *n* represents the number of the fingerprints retrieved from the database. If the copyright can be identified in* watermark verification*, *n* is 0. That is, our scheme only needs 0.24 seconds in the best case. Otherwise, our scheme needs additional 0.029 seconds for each retrieved fingerprint in the database.

Because our scheme combines watermarking and fingerprinting techniques, it can be as efficient as pure watermarking schemes and also as robust as pure watermarking schemes. A pure watermarking scheme is very efficient because it only needs to make one watermark comparison to verify the copyright. However, it may not be as robust as a pure fingerprinting scheme when dealing with images that have suffered heavy attacks.  Table 6 shows the comparison of our combined scheme and the other two pure schemes. If the copyright of the input image can be identified by* watermarking verification*, our scheme can be as efficient as pure watermarking schemes. If* watermarking verification* fails to identify the copyright, our scheme is still able to determine duplication in* fingerprinting verification*, which makes our scheme as robust as pure fingerprinting schemes.

## 5. Conclusion

This paper presented a copyright identification scheme that takes advantage of the complementary nature of digital watermarking and fingerprinting. The experimental results showed that when the watermarked image suffers moderate attacks,* watermarking verification* alone is enough to identify the copyright, and there is no need for* fingerprinting verification*. In other words, the proposed scheme can identify the copyright efficiently in this situation. On the other hand, the experimental results also showed that when the watermarked image suffers heavy attacks that render* watermarking verification* incompetent,* fingerprinting verification*, although more time consuming, can successfully determine the duplication, hence demonstrating the robustness of the proposed scheme.

One distinguishing characteristic of the proposed scheme is that it does not need a separate watermark for* watermarking verification* and a separate fingerprint for* fingerprinting verification*. The proposed scheme extracts features from the input image to generate the fingerprint, which also serves as the watermark. Hence, only one piece of information is needed for both* watermarking* and* fingerprinting verifications*.

To further improve the scheme, retrieving the stored fingerprint image from the database to restore the correct authentication logo more quickly is worth studying. When there are more than one fingerprint image in the database, the original fingerprint of the host image must be correctly retrieved; otherwise, the correct authentication logo cannot be restored. Retrieving every stored fingerprint image to restore an authentication logo for comparison is very time consuming. The scheme should provide a more efficient way that is able to find the proper one in less time, which is the future work of the research.

## Figures and Tables

**Algorithm 1:**
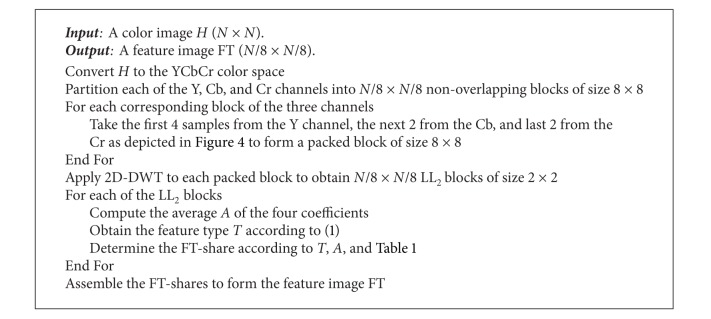
Feature extraction.

**Algorithm 2:**
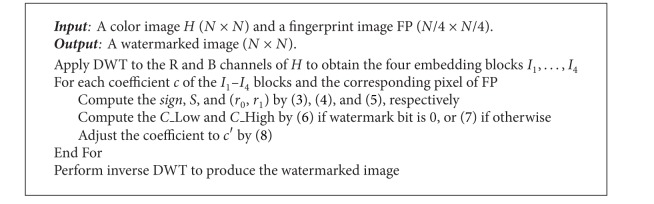
Watermark embedding.

**Algorithm 3:**
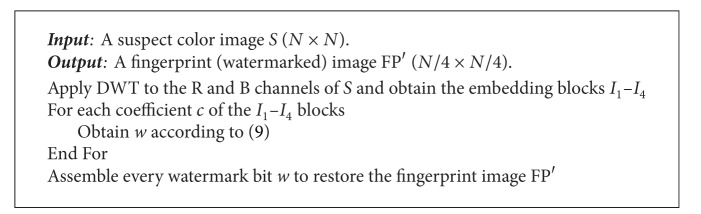
Watermark retrieval.

**Table 1:** The mapping table.

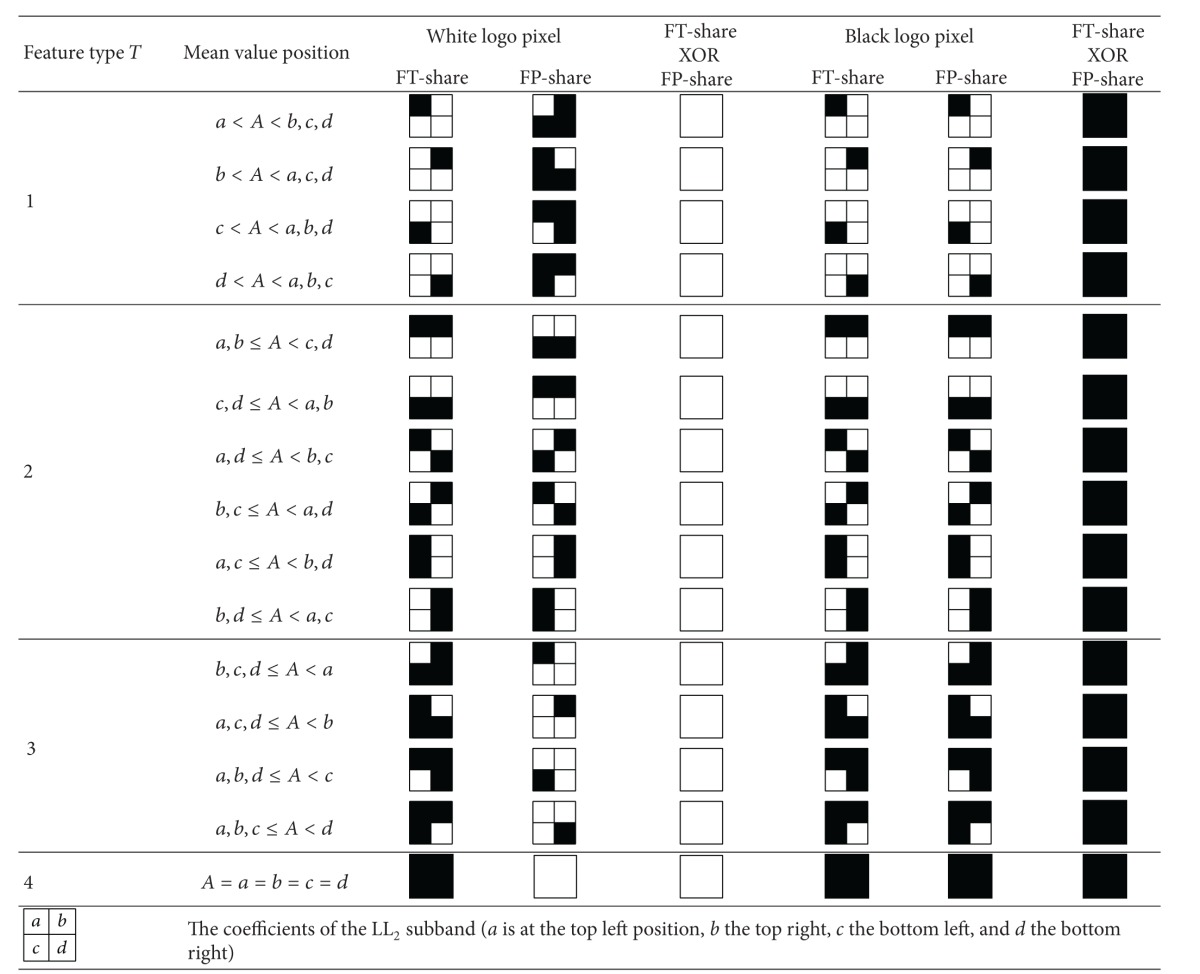

FT-share: feature share; FP-share: fingerprint share.

**Table 2:** Partial table of Table 1.

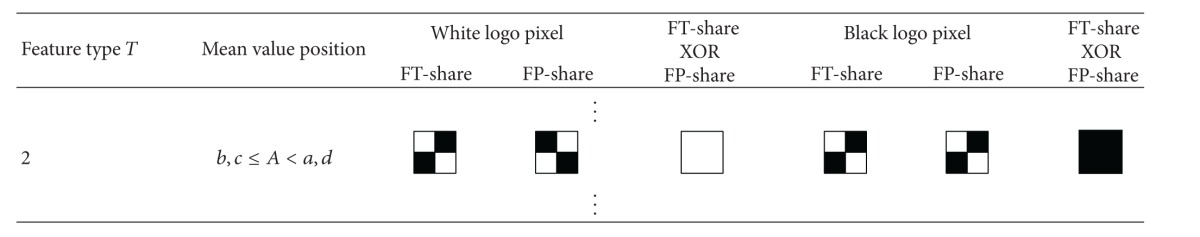

**Table 3:** The resulting AR values of the test images.

Attacks		AR (%) (Watermarking/fingerprinting)				
		Lena	Mandrill	Sailboat	Peppers	Average
None		99.93/99.93	99.76/99.76	99.98/99.98	99.63/99.63	**99.83/99.83 **

JPEG	(90)	88.31/99.71	86.84/99.44	88.04/99.88	88.16/99.49	**87.84/99.63 **
	(80)	79.76/99.73	76.17/99.02	77.29/99.58	77.91/99.12	**77.78/99.36 **
	(70)	74.58/99.46	72.12/98.73	72.61/99.54	73.05/99.17	**73.09/99.23**
	(60)	71.58/99.63	70.14/98.1	70.19/99.44	69.95/98.83	**70.47/99.00**
	(50)	71.48/99.41	70.09/97.68	70.43/99.24	69.8/98.88	**70.45/98.80**

Contrast	(10)	92.6/99.85	80.71/99.78	88.62/99.95	90.31/99.27	**88.06/99.71**
	(20)	82.86/99.76	73.12/99.56	79.27/99.78	81.81/98.68	**79.27/99.45**
	(30)	75.85/99.46	70.61/99.07	75.22/99.63	74.63/98.07	**74.08/99.06**
	(40)	72.61/99.27	70.83/97.92	72.31/99.19	72.51/98.14	**72.07/98.63**
	(50)	71.7/98.93	70.41/96.19	71.19/98.75	71.09/96.58	**71.10/97.61**
	(60)	70.39/97.68	70.65/92.7	70.95/98.1	70.53/95.8	**70.63/96.07**
	(70)	70.8/94.65	70.65/90.31	71.12/97.71	71.36/95	**70.98/94.42**

Gauss blurring	(0.5)	83.64/99.41	73.44/97.29	78.17/99.17	80.54/99.05	**78.95/98.73**
	(1.0)	76.1/98.88	70.92/95.78	74.17/98.44	73.88/98.34	**73.77/97.86**
	(1.5)	73.63/98.17	70.95/94.34	72.44/97.29	72.34/97.73	**72.34/96.88**

Scaling	*w*: 256, *h*: 256	71.66/97.92	70.75/92.53	71.22/96.83	70.73/95.8	**71.09/95.77**
	*w*: 128, *h*: 128	72.83/96.02	73.41/86.04	73.49/94.04	73.41/92.99	**73.29/92.27**

Brightness	(10)	96.66/96.78	94.41/94.92	97.85/97.85	94.46/94.46	**95.85/96.00**
	(20)	90.65/91.48	89.06/90.7	95.9/95.9	88.33/88.33	**90.99/91.60**
	(30)	85.72/87.62	83.98/86.43	93.55/93.58	83.69/83.64	**86.74/87.82**

Cropping	*w*: 480, *h*: 480	89.62/92.92	88.99/92.46	91.48/95.04	88.77/91.87	**89.72/93.07**
	*w*: 448, *h*: 448	82.71/86.82	81.71/85.67	83.79/88.89	82.03/85.82	**82.56/86.80**
	*w*: 432, *h*: 432	79.88/83.08	79.25/83.01	81.13/86.23	79/83.3	**79.82/83.91**
	*w*: 424, *h*: 432	78.88/81.76	78.49/81.96	80.52/85.86	78.59/83.06	**79.12/83.16**
	*w*: 424, *h*: 424	78.22/81.2	78/81.49	80.25/85.33	78.1/82.42	**78.64/82.61**

**Table:** (a) Lena

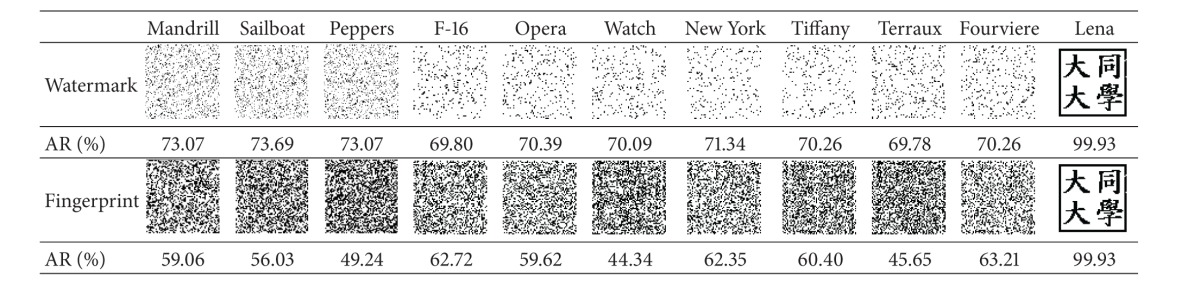

**Table:** (b) Mandrill

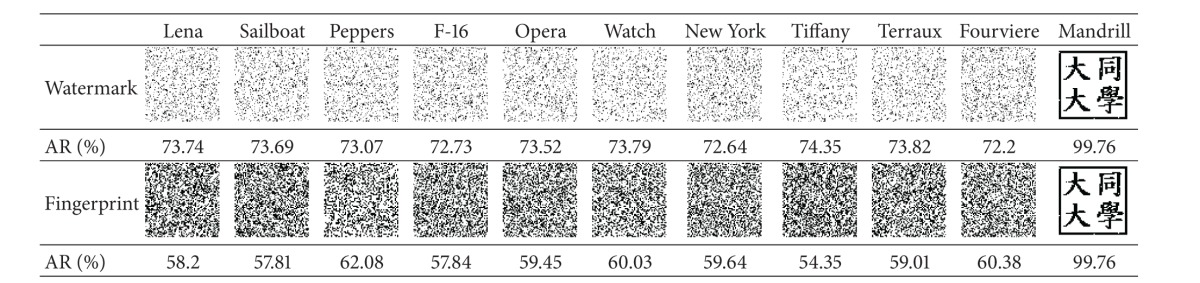

**Table:** (c) Sailboat

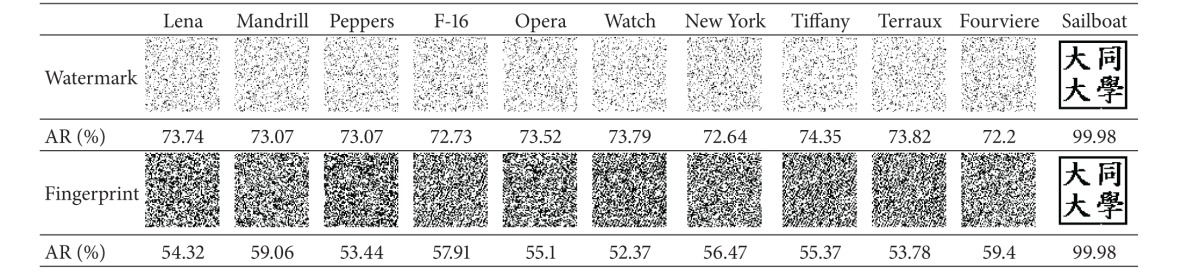

**Table:** (d) Peppers

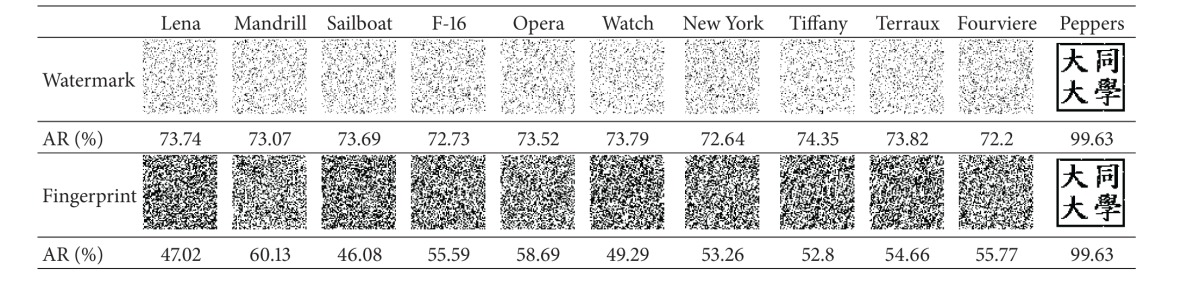

**Table 5:** The processing time (ms) for watermark extraction and logo restoration and comparison.

Stage	Lena	Mandrill	Sailboat	Peppers	Average
Watermark extraction	218	209	200	213	210

Logo restoration and comparison	20	29	39	28	29

Watermark extraction + logo restoration and comparison	238	238	239	241	239

**Table 6:** Comparison of our combined scheme and the other two pure schemes.

	Pure watermarking scheme	Pure fingerprinting scheme	Our combined scheme
Robustness	△	◯	◯
Efficiency	◯	△	◯

*◯: good; △: fair.
